# Editorial: Emerging talents in human neuroscience: Cognitive neuroscience 2022

**DOI:** 10.3389/fnhum.2023.1171860

**Published:** 2023-03-15

**Authors:** Sébastien Hélie

**Affiliations:** Department of Psychological Sciences, Purdue University, West Lafayette, IN, United States

**Keywords:** emerging talent, human neuroscience, cognitive neuroscience, editorial, Research Topic

Graduate students and postdoctoral scholars are essential to the success of academic research (Trapani, 2021). They contribute to all phases of research, from data collection, to data analysis, to the development of new methods and theories (Editorial, 2019). As a result, students often participate in the dissemination of scientific work either as authors or co–authors. Kan et al. (2021) surveyed the extent of student contributions to medical journals and found that about 15% of authors in *JAMA Internal Medicine* are students, and that this number is rising quickly.

In this Research Topic of *Frontiers in Human Neuroscience*, we highlight the work of emerging talents in human neuroscience. To feature the various types of work involving students in academic laboratories, the Research Topic includes a review article, a case report, and two original research articles.

First, Hurst and Boe reviewed various existing theories of motor imagery. Specifically, motor imagery refers to the mental rehearsal of actions without actually performing the actions. The review focused on five popular theories, namely motor simulation theory, motor emulation theory, the motor–cognitive model, the perceptual-cognitive model, and the effects imagery model. Key differences and similarities between the theories were identified, and the authors proposed testable ways to distinguish between the theories related to the locus of experienced imagery, the primary learning mechanism, the modalities involved, and the functional equivalence with actions.

Second, Maesawa et al. report their findings in treating a 77 year–old patient with Holmes tremor using deep brain stimulation (DBS). Holmes tremor is a combination of rest, action, and postural tremors that has been linked to cerebellar damage (Puschmann and Wszolek, 2011). The patient first underwent thalamotomy to target the cerebello–thalamo–cortical pathway, but the intervention failed to control tremors. The authors report that DBS applied to the subthalamic area and thalamus produced improvement of symptoms for 2 years following surgery. They therefore suggest that DBS may be a promising avenue for the control of Holmes tremor.

Third, Ulrich et al. explored the role of the right anterior insula in mental flow. Mental flow is an enhanced salience for goal–directed behavior. The experiment was performed using functional connectivity analyses of functional magnetic resonance imaging (fMRI) data. Flow conditions were compared with conditions of boredom and overload. The results show increased coupling of the right anterior insula with bilateral dorsolateral prefrontal cortex, in line with the right anterior insula's role in conditions with different salience.

Lastly, Novin et al. proposed a model of saliency of visual features that relies on the structure of the visually presented image. The model integrates features at multiple scales and can be used to account for human data. The results shed new lights on how image properties can drive visual attention and the proposed mechanisms can be used to improve existing models of visual attention.

The four articles included in this Research Topic highlight the importance of emerging talents in human neuroscience to organize knowledge, improve the transfer of knowledge to better treat patients, and learn about how the brain processes information using both neuroimaging and computational modeling. While this Research Topic only provides a glimpse of the amazing work and talent of emerging neuroscientists, we hope that it will make current neuroscientists optimistic about the future of neuroscientific research. The field is in good hands!

## Author contributions

The author confirms being the sole contributor of this work and has approved it for publication.
